# A small native predator reduces reproductive success of a large invasive fish as revealed by whole-lake experiments

**DOI:** 10.1371/journal.pone.0214009

**Published:** 2019-04-03

**Authors:** Joshua R. Poole, Przemyslaw G. Bajer

**Affiliations:** 1 University of Minnesota, Minnesota Aquatic Invasive Species Research Center, St. Paul, Minnesota, United States of America; 2 Idaho Fish and Game, McCall, Idaho, United States of America; Uppsala Universitet, SWEDEN

## Abstract

The extent to which native fish communities might control the success of invasive fish has been of interest to ecologists, but it has been rarely addressed using experiments. We conducted an experiment in six small lakes in the Upper Mississippi Region to test the effects of a small native predator, bluegill sunfish (*Lepomis macrochirus)* on the recruitment of a large, invasive fish, the common carp (*Cyprinus carpio*). Bluegills are predominant throughout the region and were previously shown to consume carp eggs and larvae. We stocked both lakes at each of our 3 sites with adult carp (spawners) and one lake at each site with bluegills. We repeated the experiment at two of the three sites for two consecutive years. In each lake we assessed the abundance of post-larval carp one month after spawning (backpack electrofishing surveys) and at the end of the season (mark-recapture). For each site/year combination, catch rate of post-larval carp was typically an order of magnitude higher in control than bluegill lakes, but it often declined quickly over time. The abundance of end-of-seasonal juveniles was significantly higher (no 95% CI overlap) in control lakes than in bluegill lakes, except for one pair of lakes during one year when both the control and bluegill lake had similar, low abundance of end-of-season carp. Overall, our results support the hypothesis that common carp recruitment is substantially reduced in habitats dominated by bluegills. We also suggest our results may be applicable to other species, and that managers should explore how predation on early life stages may control other invasive species.

## Introduction

The extent to which native fish communities, especially native predators, might control the establishment and success of invasive fish has been of interest to marine and freshwater ecologists [1,2,3,4]. Shedding light on this relationship might explain fish invasions at local and regional scales [5], examine the need to conserve native fish communities—many of which are severely eroded [6], and suggest management strategies to hinder invasions [2]. The hypothesis that predation might play an important role in hindering fish invasions is supported by several lines of evidence. Predation has been shown to have a strong effect on native fish communities [7,8,9]. Thus, we hypothesize that predation might have similar effects on fishes that are introduced to new habitat. Further, non-native fish become invasive in only a fraction of locales to which they are introduced, despite seemingly favorable abiotic conditions, suggesting that biotic processes such as predation might often be limiting their success [10,5] Finally, small-scale (laboratory, mesocosm, pond) experiments suggested a strong effect of native predators on at least some invasive fish [2, 11, 12]. Despite significant interest among aquatic ecologists and potential management applications, the hypothesis that native predatory fish might be instrumental in controlling invasive fish has never been tested using large-scale, natural experiments.

The extent to which native predatory fish might control invasive fish is influenced by multiple factors [11]. For small species of invasive fish, predation might occur during all life stages [2]. For fishes that attain large body size, predation might occur primarily during early development, especially if these species do not employ parental care (e.g., nest building, guarding eggs, etc.). This scenario (large fishes that do not employ parental care) applies, for example, to the several species of the invasive carps in North America [1]. Spatial and temporal aspects of life history also need to be considered. For example, invasive fish may employ seasonal migrations to spawn in ephemeral habitats where native predators are rare [13]. In other cases, invasive fish may spawn at times when predatory pressure is weakened (e.g. during spring floods) [14]. Defense mechanisms, such as production of toxins (e.g. toxic eggs, venomous spines) could also weaken the effect of predation [15].

The complexities of life histories, behaviors, habitats, and local adaptations make broad generalizations about the role of predation in controlling the success of invasive fish challenging. Nevertheless, experimental studies might help addressing key tenants of the predation-invasibility relationship in fish and might identify certain types of species or habitats where predation is likely to be important. Arguably, the case of the invasive common carp (*Cyprinus Carpio*) in the Upper Mississippi Region (UMR) is one of the best documented scenarios where native predatory fish might control the success of the invader to a significant degree [13,2,16,5,17,18]. The common carp (or ‘carp’) is a large and highly fecund fish [19]. It spawns in shallow waters where it broadcast eggs over aquatic vegetation to which the eggs adhere [19]. Carp lack parental care and both its eggs and larvae are vulnerable to predation. Field observations, lab experiments, and mesocosm experiments have suggested that the survival of carp eggs and larvae in UMR can be severely limited by a small native predatory fish, the bluegill sunfish (*Lepomis macrochirus)* that forages on carp eggs and larvae [13,2,16,5,18]. This hypothesis was proposed to explain the spatial pattern within UMR where carp recruitment occurred only in bluegill-free habitat, such as shallow lakes and marshes that experience winter fish kills due to hypoxia (bluegills are sensitive to hypoxia), whereas carp recruitment very rarely occurred in lakes that contained bluegills [13,5]. It also seemed to explain the pervasive spawning migrations of adult carp in the UMR out of deep lakes where bluegills are often abundant and into peripheral shallow marshes where bluegills are often absent [5,20]. In addition to elucidating fundamental invasion mechanisms, this hypothesis has clear management implications. Specifically, that if hypoxia could be prevented in some winterkill-prone marshes to stabilize bluegill populations, those landscapes may no longer function as carp nurseries.

Despite many pieces of evidence, the hypothesis that bluegill sunfish might control carp recruitment has not been tested using whole-lake experiments. In this study, we conducted a two-year experiment where adult carp were stocked into six small natural lakes, and bluegill were stocked into half of these lakes. The recruitment of carp was then assessed in each lake at two stages: post-larval juveniles (first stage where carp had outgrown bluegill predation) and end-of summer juveniles (fish that recruit into population). Our results shed a new light on the predation-invasibility hypothesis for broadcast-spawning invasive fish in regions dominated by small predators. It also suggests management recommendations specific to carp populations within the UMR.

## Materials and methods

### Study lakes and experimental design

This study consisted of an experiment conducted in small lakes over two consecutive years. It included four lakes in 2016, which were used again in 2017 as well as two more lakes that were added in 2017 (Table 1). The lakes ranged in size from 0.35 ha to 1.8 ha and had maximum depths of < 3 m (Table 1). All lakes were located within 100 km from the Twin Cities metro area in Minnesota, USA: Crown College lakes (44.884956, -93.745116); Albert Lea lakes (43.669828, -93.386296); Metro treatment lake (44.702426, -93.488563), Metro control lake (45.114023, - 93.184685). Two lakes were present at of each three sites (Crown College, Albert Lea, Metro), allowing a paired design (treatment and control at each site). Lakes at Crown College and Albert Lea sites were directly adjacent, while Metro site lakes were both located in the Twin Cities area, approximately 30 km apart. No connections passable to fish existed between the pairs of lakes, except in Crown College, where both lakes were connected with a narrow (~ 2 m wide, 0.5 m deep) channel of stagnant water heavily overgrown with emergent vegetation. We placed three panels of small mesh (~ 3 mm mesh size) nets to prevent fish migrations between these two lakes. Net bottoms were weighed down using sand bags while tops were held above water using metal posts. Lakes were used after obtaining permission from owners (Shell Rock River Watershed District, Crown College, the City of Shoreview, and Prior Lake Watershed District). All work was in accordance with special permit 22991A from the Minnesota Department of Natural Resources.

**Table 1:** Physical characteristics of the study lakes, including the biomass of bluegills stocked at the beginning of the experiment, the number of carp males and females stocked each year, and the estimated number of carp eggs per hectare. Lakes that were not stocked with bluegills were used as controls. TP is total phosphorus. *C*. *demersum is Cerathophylum demersum*.

Site	Treatment	Year	Bluegill biomass (kg/ha)	Carp M/F	Carp eggs (10^6^/ha)	Area (ha)	Max depth (m)	TP (ug/l)	Dominant vegetation	Vegetative cover (%)
Crown College	Bluegill	2016	106.1	9/9	18.0	0.85	1.6	86.5	*C*. *demersum*	63.8
Crown College	Control	2016	0.0	5/5	24.0	0.35	1.4	123.3	*C*. *demersum*	42.5
Albert Lea	Bluegill	2016	104.3	20/11	23.5	0.65	1.6	47.7	*Elodea spp*.	70.5
Albert Lea	Control	2016	0.0	26/14	21.0	1	1.7	97.0	*Elodea spp*.	92.0
Crown College	Bluegill	2017	104.0	15/10	19.4	0.85	1.6	88.6	*C*. *demersum*	26.0
Albert Lea	Bluegill	2017	105.8	14/8	19.2	0.65	1.6	120.0	None	>1%
Albert Lea	Control	2017	0.0	24/16	20.0	1	1.7	293.7	None	>1%
Metro	Bluegill	2017	107.3	9/7	20.6	0.55	1.5	87.0	*Elodea spp*.	3.2
Metro	Control	2017	0.0	29/26	20.6	1.8	2.3	33.7	*C*. *demersum*	58.3

We strived to conduct our experiment in lakes that initially were fishless but such systems could not be found and some of our lakes contained low numbers of native species that often occur in small, hypoxia-prone lakes such as fathead minnows (*Pimephales promelas)* and black bullheads (*Ameiurus melas)* (S1 Table). All of our study lakes were eutrophic to hypereutrophic, which was determined by collecting water samples monthly from May-July and analyzing them for total phosphorus (Table 1). The lakes’ dissolved oxygen level, temperature, water clarity, zooplankton, and aquatic vegetation cover were also monitored (Table 1; S1 Fig). Overall, our lakes were representative of small, shallow, productive systems within the UMR that carp often use as nurseries (Bajer et al. 2015). To conduct the experiment, all lakes were stocked with adult carp, while every other lake (one in each pair) was also stocked with bluegills (see below).

### Stocking study lakes with bluegills and adult carp

Bluegill sunfish were collected from nearby lakes using trapnets (front frame 0.7 m tall and 1 m wide followed by four hoops 0.6 m in diameter, 1 cm mesh; Duluth Net Co. Duluth, MN) in April and May of each year and transported to one lake at each experimental site (the other lake at each site was used as a control). Bluegills were stocked at a density of approximately 100 kg/ha (Table 1), a previously-reported density for bluegills in the littoral zone (where carp spawn) of eutrophic lakes of the UMR [21,1]. The status of bluegill population in each lake (catch-rate, survival, recruitment) was monitored by conducting trapnet surveys and mark-recapture analyses at the end of the season. Two trapnet surveys were conducted in each lake, using 4 nets set overnight. The surveys occurred in late August and September of each year. Bluegills caught in each survey were counted to calculate mean catch per effort (bluegills/trap), marked (fin clip) and released to calculate population abundance using mark-recapture analyses. Captured bluegills were divided into two categories: adults (bluegills that were stocked) and juveniles (in 2016 those were age-0 bluegills that recruited within each lake; in 2017 those were a mixture of age-1 bluegills from 2016 and age-0 bluegills from 2017).

Adult common carp were collected from nearby lakes using boat electrofishing and stocked into all experimental lakes approximately two weeks after bluegills were stocked, to allow time for bluegills to acclimate before the carp began their spawning activity. All stocked carp were in pre-spawning condition. The gender of carp was determined visually (females had extended abdomens due to large ovaries; males readily expressed sperm). Each carp was measured and the number of eggs per female was estimated from the total body length [1]. The number of females stocked into each lake, between 10 and 15 per lake, was calculated to achieve a theoretical propagule pressure of 20 million eggs/ha egg density at which carp recruitment is often highest [1]; i.e. we wanted to reduce the risk of recruitment failure due to low propagule pressure. Male carp were stocked at a ratio of approximately 1–2 per female (Table 1).

For lakes that were re-used in 2017, removal of carp and bluegills at the end of the 2016 season to establish “fishless” conditions for the 2017 season was not feasible, thus, carp and bluegills were left in lakes over the winter and the same treatments continued in each lake in 2017 (Table 1). For bluegill lakes that were used in 2016 and then re-used in 2017, mark-recapture estimates were conducted in the spring of 2017 to estimate the overwinter survival and recruitment of bluegills to determine how many new bluegills needed to be stocked in 2017 to achieve the biomass of 100 kg/ha (S2 Table). Overwinter survival of carp could not be assessed (adult carp were notoriously difficult to sample), thus adult carp were stocked in 2017 at the same density as in 2016. Animals were handled according to the University of Minnesota Animal Care Protocol 1601-33424A).

### Abundance of post-larval carp

To quantify the effect of bluegills on the reproductive success of carp, we estimated the abundance of post-larval carp in each lake. Those carp were approximately 1–2 months old and 30–60 mm in length. At such size, the carp are no longer vulnerable to bluegill predation (i.e. they well exceeded bluegills gape size [22]). Thus, we considered this life stage as one that most accurately portrayed the direct effect of bluegill predation on carp recruitment. We estimated the abundance of post-larval carp by conducting backpack electrofishing surveys that commenced one month after the carp were observed spawning and were conducted approximately every two weeks after that for approximately two months. Three such surveys were conducted in each lake in 2016, and four in 2017. Each survey consisted of four 15-min transects. In each transect, we waded slowly along the shoreline of the lake (carp spawn near shore and post-larval juveniles typically occupy littoral areas) and electrofished in zigzagging motion. Carp and all other stunned fish were netted and counted. Total length was measured for the first 50 individuals of each species. A mean catch-per-unit-effort (CPUE; number of post-larval carp per hour) was calculated for each survey day, by averaging CPUEs among transects. Next, mean CPUE for each lake in each year was calculated. Mean annual CPUEs were then analyzed using a nested general linear model to test whether CPUE was affected by treatment type, taking into account that a pair of treatments (bluegill and control) was present at each site. Year effect was not included due to small sample size as necessitated by the whole-lake nature of our experiments (the overall year effect, when tested independently, was not significant).

### Abundance of end-of-season young-of-year carp

We also quantified the abundance of end-of-season young-of-year (YOY) carp in each lake. At this stage, carp were 4–6 months old and approximately 80–160 mm in length. While no longer directly influenced by bluegill predation, this life stage is of management importance because these juveniles are considered large enough to recruit into the next year class. Abundance of YOY carp (the number of individuals per hectare) was assessed using mark-recapture analyses. To facilitate those analyses, each lake was surveyed with small-mesh trapnets. During each survey, five trapnets were placed in the littoral zone of each lake overnight. Captured carp were marked with a fin clip (unique clips were used on each sampling occasion), and released. The population and 95% CI were estimated using the multiple-census Schnabel method in which the proportion of marked fish is examined during each consecutive census [23]. The calculations were conducted using FSA package in R [24,25]. To analyze the results statistically, we examined whether the 95% confidence intervals overlapped between the control and bluegill lakes within each site-year combination. In most lakes, three or four surveys yielded more than 20 recaptures, which was deemed sufficient for population estimates. In other lakes, we conducted up to 11 surveys due to low recapture rates (Table 2). In lakes where no carp were captured in the first three surveys, the population was assumed to be zero; those lakes typically also had no post-larval carp captured. In some lakes (Crown College), we also conducted supplementary beach seining (30 wide, 3 m tall, 1 cm mesh; Duluth Net Co. Duluth, MN) to increase catch rates for mark-recapture analyses.

**Table 2:** Mark-recapture estimates for the abundance (individuals per hectare) of the end-of-season YOY carp in each study lake. The lakes were either stocked with bluegills or not (controls). In most lakes, three or four surveys yielded more than 20 recaptures, and surveying was deemed complete. In some cases, where recapture rates were low, sampling continued for up to 11 surveys. In lakes were no YOY carp were captured in the first three surveys, the population was assumed to be zero.

Location	Year	Treatment	No. surveys	Total marked	Total recaptured	Density (ind./ha)	95% CI
Crown College	2016	Bluegill	11	109	7	1,090	583–2,358
Crown College	2016	Control	8	230	14	6,973	4,331–13,057
Albert Lea	2016	Bluegill	3	842	21	4,449	1,992–6,584
Albert Lea	2016	Control	3	1,084	30	31,031	22,425–46,270
Crown College	2017	Bluegill	3	0	0	0	0
Albert Lea	2017	Bluegill	4	307	149	1,126	634–1,933
Albert Lea	2017	Control	7	184	21	888	590–1,396
Metro	2017	Bluegill	3	0	0	0	0
Metro	2017	Control	4	126	36	1,336	984–1,860

## Results

### Abundance of post-larval carp

**Fig 1:**
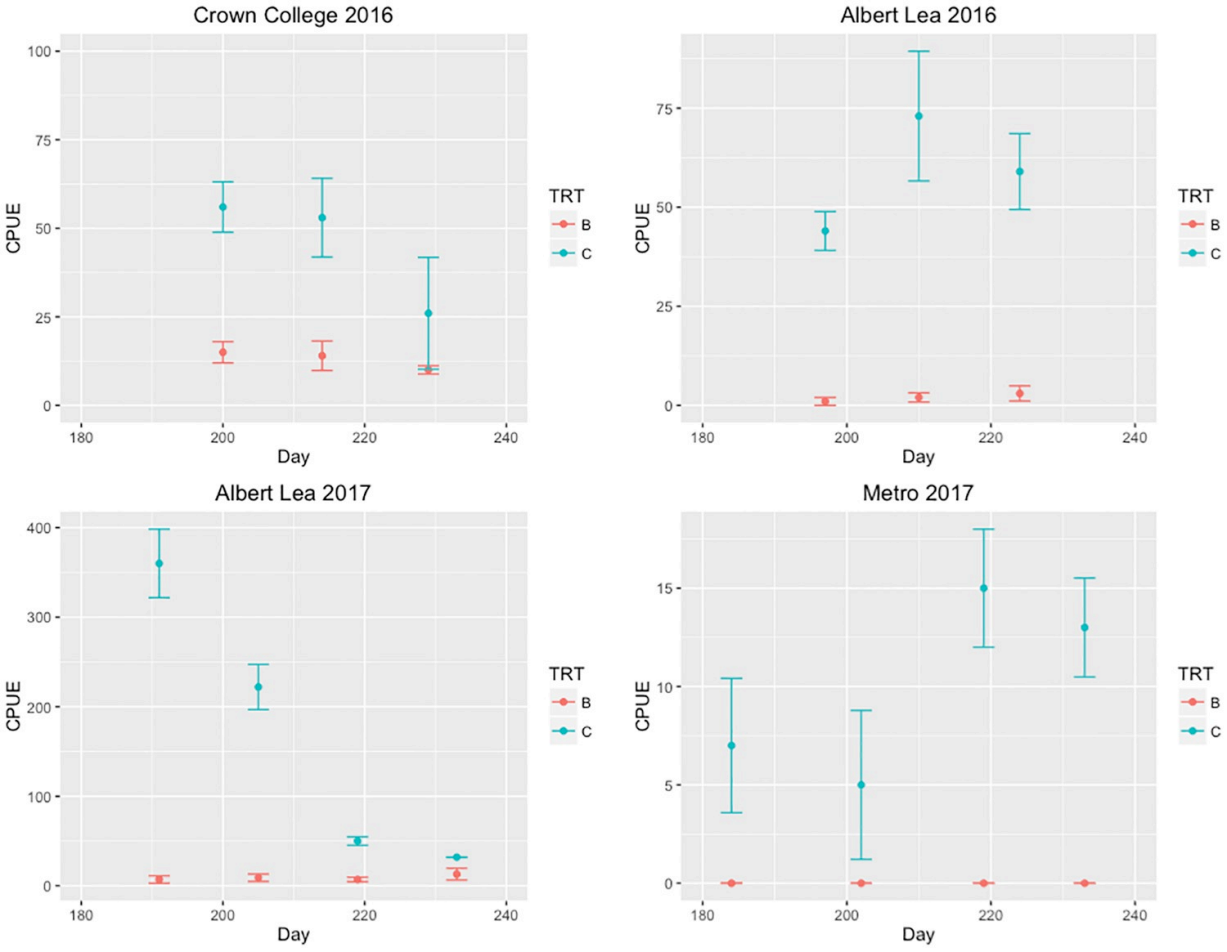
Catch rate of post-larval carp caught per one hour of backpack electrofishing in the study lakes stocked with bluegills (B) or in control lakes (C) in 2016 and 2017. A pair of bluegill and control lakes was located at each of three sites: Albert Lea, Crown College and Metro. Metro lakes were only used in 2017. The control lake in Crown College in 2017 was invaded by bluegills and was not used in the analysis or included in this figure. CPUEs of post-larval carp were consistently higher in control than treatment lakes at each site (*df* = 1; *F* = 6.57; *P* = 0.09).

**Fig 2:**
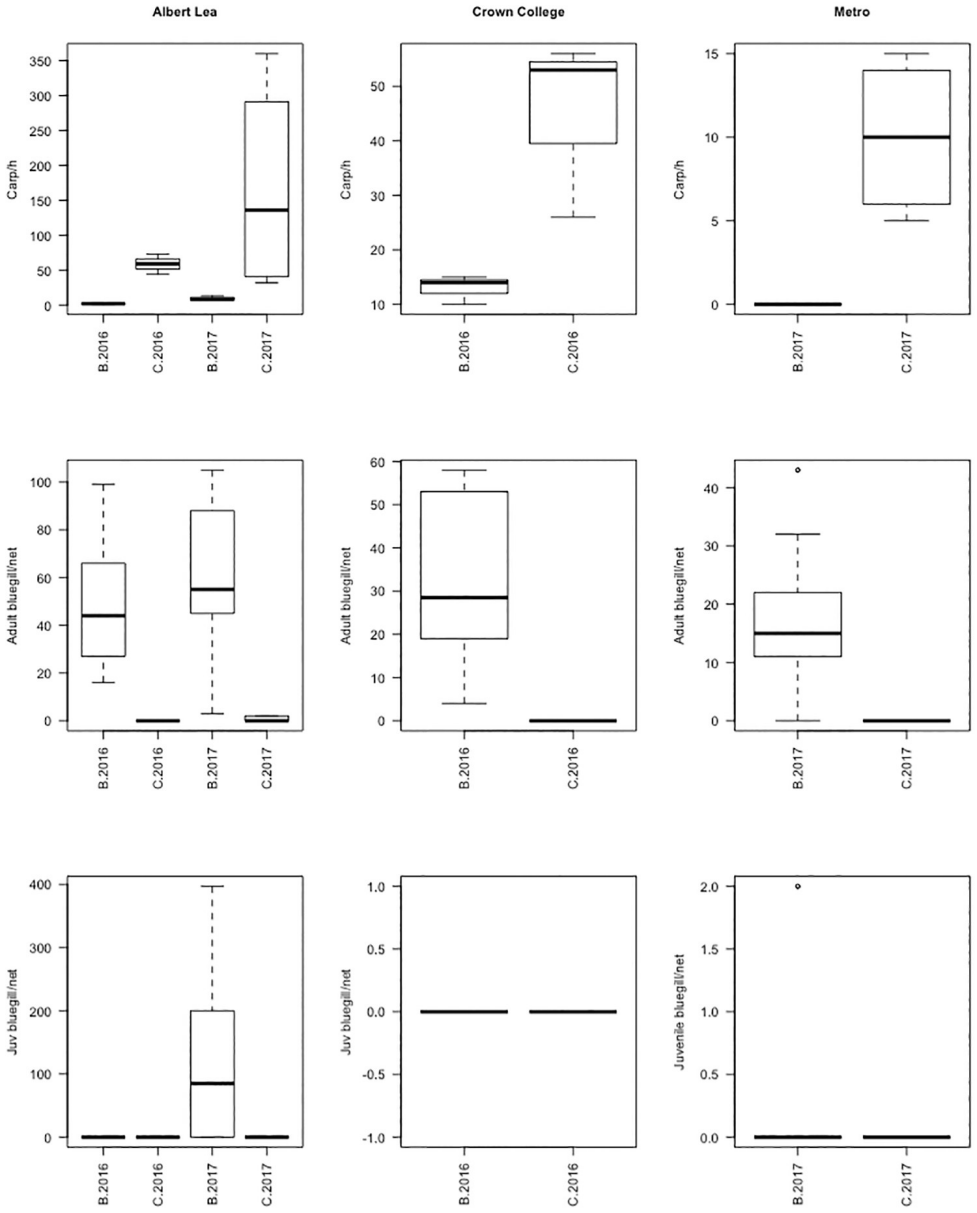
Catch rates of post-larval carp (top row), adult bluegills (middle row) and juvenile bluegills (bottom row) in the study lakes. Juvenile bluegills in 2016 were young of the year. Juvenile bluegills in 2017 were mixture of age-1 and young of the year.

CPUEs of post-larval carp were consistently higher in control than treatment lakes at each site. The effect was clear but not significant, likely due to low sample size (*df* = 1; *F* = 6.57; *P* = 0.09; Figs 1 and 2). Further, in three of the five lakes stocked with bluegills, no post-larval carp were captured (Figs 1 and 2). One of the control lakes (Crown College in 2017) was invaded by bluegills and was excluded from the analyses (S2 Fig). When averaged across the sampling dates within each year, the mean CPUEs of post-larval carp were higher, by at least a factor of three, in control lakes than in lakes stocked with bluegills (Fig 2).

### Abundance of end-of-season juvenile carp

The abundance of YOY carp at the end of the season was significantly higher (no overlap between 95% CIs) in control lakes than in bluegill lakes in three of the four year and site combinations (Albert Lea 2016, Crown College 2016, Metro 2017; Table 2), and not statistically different in one (Albert Lea in 2017) where the density of YOY carp was low in both the bluegill and control lake (Table 2). The control lake in Crown College in 2017 was invaded by bluegills and was not used in the analysis. Across the years and sites, mean and median YOY carp density in control lakes were 10,455 ind./ha and 6,973 ind./ha, respectively. Mean and median YOY density in lakes stocked with bluegills were 1,333 ind./ha and 1,090 ind./ha, respectively.

## Discussion

This, to our knowledge, is the first whole-lake experiment to test the effect of a native predator on the recruitment of an invasive fish. Our results indicate that common carp’s recruitment in UMR is likely to be reduced by several folds in environments with abundant populations of bluegills sunfish. This conclusion is based on the fact that both the catch rate of post larval carp and the density of end-of-season YOY carp were consistently lower in lakes stocked with bluegills than in control lakes. While our results were clear, they were not always statistically significant, likely due to small sample size necessitated by the whole-lake nature of this experiment. Though our experiment represents relatively transient conditions, empirical evidence from other systems suggests that bluegills can affect a long-term and broad ranging control of carp recruitment. For example, in Lake Susan and in the Phalen Chain of Lakes in Minnesota, bluegills appear to have been controlling carp recruitment for over 10 years [5,20]. Further, surveys of several hundreds of lakes across Minnesota showed very low occurrence of carp recruitment in lakes dominated by bluegills over the last 20 years but high frequency of recruitment in systems where bluegills were sparse [12].

The field of biological invasions often focuses on using large, top predators to control invasive fish [15]. Our work shows that small, native predators that forage on eggs and larvae of the invader might also be important. Small predators might even be more effective than the large predators, because they tend to be more abundant [11]. Predation on eggs and larvae has been shown to strongly affect recruitment rates of marine and freshwater fishes [26,27] and we propose that this process be more broadly considered while explaining the recruitment of invasive fish. Further, life history reversal often occurs in fish, where small omnivorous prey species might control large predators during the predator’s early development by foraging on their eggs and larvae [28]. Thus, the potential spectrum of biocontrol agents that affect the invader might be broad and might include species that are not intuitively considered as “predators”. We hypothesize that the biocontrol scenario described in this study may be applicable to a wide range of invasive fishes, particularly those that broadcast spawn or provide no parental care, which is common in bony fishes; only ~20% of families of bony fishes contain species that employ parental care [29,30,31]. We encourage applied ecologists and managers from regions outside the UMR to consider whether native fishes from their particular regions, and especially small predators that forage on early life stages, might be capable of biocontrol on introduced fishes.

While we were unable to determine exactly how bluegills suppressed the recruitment of carp, we assumed that predation played a major role because prior experiments have shown that bluegills forage heavily on carp eggs and larvae [1,16,12]. Directly monitoring the predation of bluegills on carp eggs and larvae during this experiment would have been challenging and potentially disrupting. In our lakes, carp spawning lasted only a few days and conducting extensive surveys of bluegill populations to examine their diets for presence of carp eggs would have likely disrupted the predation process, which we wanted to avoid. We suspect that other processes, including competition for planktonic food resources, did not play a significant role in regulating carp recruitment in our experiment. Larval common carp heavily rely on cladocerans during early development [32]. Because bluegills are also known for foraging on cladocerans, they might compete with larval carp for food [33, 34]. However, in our experiment, cladoceran densities were generally similar or higher in lakes stocked with bluegills than in control lakes (S1 Fig), thus it is unlikely that bluegills controlled carp recruitment via competition for the same planktonic resources. That being stated, our conclusions regarding the potential effect of zooplankton communities on carp recruitment are tentative because our experiment represented transient conditions. In real world scenarios, we expect competition for planktonic resources to be much stronger in lakes with established bluegills (and fish in general) communities than in seasonally unstable and relatively fishless habitats where carp often recruit in large numbers (e.g. lakes that winterkill). We hypothesize that availability of planktonic resources may have secondary effect, in addition to predation, on carp recruitment.

Our results suggest that bluegills had an especially strong effect on carp recruitment in the early, post-larval stages, but it appears that later stages of recruitment were affected by other processes. In Albert Lea in 2017, capture rates of post-larval carp were initially over 200 times higher in the control lake than in the bluegill lake, but at the end of the season, both lakes had similar (low) abundance of YOY carp. At the end of the season, YOY in the control lake were emaciated, small (~ 30–50 mm, while YOY carp in the bluegill lake were ~ 150 mm) and most had multiple external parasites (*Lernaea spp*.; anchor worm). The initially high density of post-larval carp in the control lake in Albert Lea in 2017 in combination with the presence of yearling carp from previous year (which we were unable to remove at the end of the 2016 season) and the high density of adult carp (stocked in 2016 and then in 2017), might have resulted in increased competition for food and increased transmission of parasites. Negative effects of high density on carp recruitment (i.e. density dependence) have been also shown by others [35,36], but the process by which density dependence occurs has been poorly documented. Our anecdotal observations of the poor physical condition of the YOY carp in the high-density control lake and the presence of parasites on them suggest that malnutrition and pathogen transmission might play an important role in density-dependent recruitment in common carp.

Our study has direct management implications for ecosystems within the Mississippi River Drainage where bluegills are native. It suggests that within this region, enhancing bluegill populations where carp spawn might hinder carp invasion. Since winter hypoxia is a major driver of bluegill abundance in UMR [37] and since carp often exploit hypoxia-prone marshes where bluegills are few as nursery areas [1], stabilizing bluegills in hypoxia-prone habitats should be considered. Winter aeration has been shown to be effective at averting winterkills and enhancing bluegill survival in some hypoxia-prone habitats [38]. For example, winter aeration has been used in Lake Susan in UMR to prevent winterkills and curb carp recruitment for more than two decades [13]. Researchers [38] found, however, that aeration was less effective in waterbodies shallower than 1.5 m, thus other methods to stabilize bluegills should be examined. For example, such areas might need to be blocked off with barriers or deterrence systems to prevent carp from exploiting them as nurseries. We do not recommend introducing bluegills into areas where they are not native to control carp. Instead, native species that might perform this kind of control should be examined [39]. In particular, habitats where native species might be sparse as a result of seasonal instability or disturbance events such as floods, disease outbreaks, pollution, or hypoxia should be examined, as such habitats may function as carp recruitment hotspots. Our results also provide useful empirical data on the specific numbers of carp recruits produced in different habitat types, which might increase biological realism of carp population models [35,36,40,41,18] to further aid carp management.

## Supporting information

S1 Table Backpack electrofishing catch-per-unit-effort (CPUE) for non-target species in each experimental lake. C stands for control lakes and B stands for lakes stocked with bluegills. The control lake at Crown College in 2017 was invaded by bluegills and was not used in the analysis. NA is used to denote that Metro lakes were not used in 2016.
(DOCX)

S2 Table Number of adult bluegills stocked into each treatment lake in 2016 and 2017 and mark-recapture estimates for the number of adult bluegills remaining in each lake at the end of the season. The abundance of juvenile bluegills (age-0 and age-1) was also estimated using mark-recapture (in 2017) or catch rates in relation to catch rates of adult bluegills (2016). For the estimates derived using catch rates, 90% CI were not available.
(DOCX)

S1 Fig Zooplankton in experiments lakes. Samples were collected in May and June. AL = Albert Lea, CC = Crown College, MET = Metro. Metro lakes were not used in 2016. Zooplankton was collected from two sites in each lake during May and June approximately every 2 weeks using a WILDCO^®^ Wisconsin Plankton sampling net with a 20-μm mesh. Zooplankton was collected from a boat using vertical tows from a depth of 1m.
(TIF)

S2 Fig Catch rates of post-larval carp (top row), adult bluegills (middle row) and juvenile bluegills (bottom row) in the Crown College lakes in 2017. The control lake was invaded by bluegills (note the bluegill presence in the control lake), and was therefore excluded from analyses.
(TIFF)

S1 Dataset Backpack electrofishing capture rates for 2016 and 2017. (TXT)
