# Supplementary material for: A small native predator reduces reproductive success of a large invasive fish as revealed by whole-lake experiments
Source: PLoS One. 2019 Apr 3;14(4):e0214009. doi: 10.1371/journal.pone.0214009 (PMC6447168; doi:10.1371/journal.pone.0214009)
Supplement: S1 Table — C stands for control lakes and B stands for lakes stocked with bluegills. The control lake at Crown College in 2017 was invaded by bluegills and was not used in the analysis. NA is used to denote that Metro lakes were not used in 2016. (DOCX) [file pone.0214009.s001.docx]

|  | Fathead Minnow | | Black Bullhead | | Mud Minnow | | Golden Shiner | | Largemouth Bass (Juvenile) | | | Green Sunfish | | |
| --- | --- | --- | --- | --- | --- | --- | --- | --- | --- | --- | --- | --- | --- | --- |
|  | 2016 | 2017 | 2016 | 2017 | 2016 | 2017 | 2016 | 2017 | 2016 | 2017 | 2016 | | 2017 |  |
| Crown College C | 57.80 | 75.50 | 5.00 | 6.33 | 2.67 | 4.50 | 0.00 | 0.00 | 0.00 | 0.00 | 0.00 | | 0.00 |  |
| Crown College B | 29.10 | 73.67 | 8.50 | 6.67 | 5.33 | 13.00 | 1.00 | 1.00 | 0.00 | 0.00 | 0.00 | | 0.00 |  |
| Albert Lea C | 0.00 | 0.00 | 0.00 | 0.00 | 0.00 | 0.00 | 0.00 | 0.00 | 0.00 | 0.00 | 0.00 | | 0.00 |  |
| Albert Lea B | 0.00 | 0.00 | 0.00 | 0.00 | 0.00 | 0.00 | 0.00 | 0.00 | 0.00 | 0.00 | 0.00 | | 0.00 |  |
| Metro C | NA | 81.75 | NA | 0.00 | NA | 0.00 | NA | 0.00 | NA | 0.00 | NA | | 22.00 |  |
| Metro B | NA | 0.25 | NA | 0.50 | NA | 0.00 | NA | 0.00 | NA | 45.75 | NA | | 0.00 |  |
