# Supplementary material for: A small native predator reduces reproductive success of a large invasive fish as revealed by whole-lake experiments
Source: PLoS One. 2019 Apr 3;14(4):e0214009. doi: 10.1371/journal.pone.0214009 (PMC6447168; doi:10.1371/journal.pone.0214009)
Supplement: S2 Table — The abundance of juvenile bluegills (age-0 and age-1) was also estimated using mark-recapture (in 2017) or catch rates in relation to catch rates of adult bluegills (2016). For the estimates derived using catch rates, 90% CI were not available. (DOCX) [file pone.0214009.s002.docx]

| Location | Year | Size (ha) | Adult bluegills stocked at the beginning of season | Number of mark-recapture surveys | Total marked | Total recaptured | Estimated number of adult bluegills at the end of season | Estimated adult bluegill density at the end of season (ind./ha) | 90% CI  adult bluegill density (ind./ha) | Estimated juvenile bluegill density at the end of the season (ind./ha) | 90% CI  juvenile bluegill density (ind./ha) |
| --- | --- | --- | --- | --- | --- | --- | --- | --- | --- | --- | --- |
| Crown College | 2016 | 0.85 | 1615 | 3 | 178 | 40 | 581 | 684 | 565 - 864 | 1,547 | - |
| Albert Lea | 2016 | 0.65 | 1276 | 3 | 252 | 87 | 548 | 844 | 644 - 1226 | 4,792 | - |
| Crown College | 2017 | 0.85 | 1011 | 2 | 167 | 24 | 531 | 625 | 449 - 891 | 6,169 | 4378 - 8927 |
| Albert Lea | 2017 | 0.65 | 642 | 3 | 313 | 139 | 658 | 1012 | 831 - 1295 | 6,855 | 4820 - 9406 |
| Metro | 2017 | 0.55 | 1095 | 3 | 190 | 43 | 547 | 995 | 775 - 1295 | 229 | 80 - 455 |
