# Supplementary figures and images for: A small native predator reduces reproductive success of a large invasive fish as revealed by whole-lake experiments
Source: PLoS One. 2019 Apr 3;14(4):e0214009. doi: 10.1371/journal.pone.0214009 (PMC6447168; doi:10.1371/journal.pone.0214009)

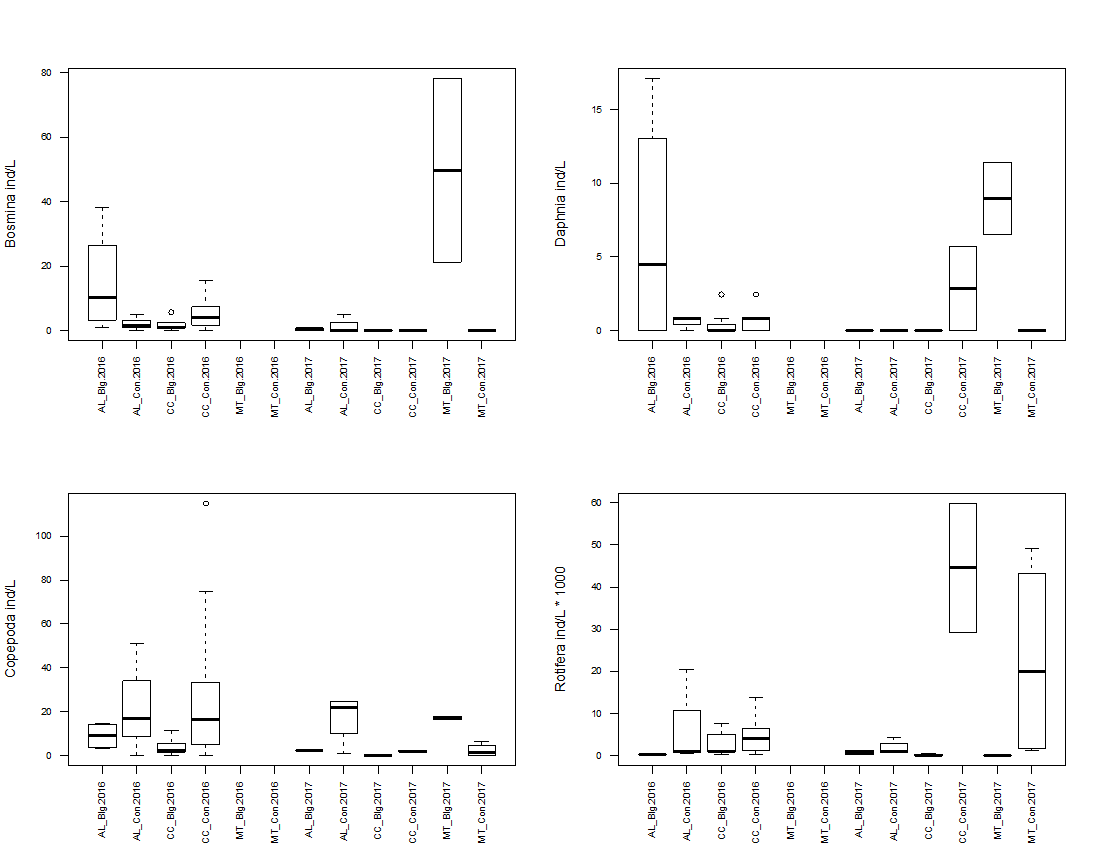

Supplement: S1 Fig — Samples were collected in May and June. AL = Albert Lea, CC = Crown College, MET = Metro. Metro lakes were not used in 2016. Zooplankton was collected from two sites in each lake during May and June approximately every 2 weeks using a WILDCO® Wisconsin Plankton sampling net with a 20-μm mesh. Zooplankton was collected from a boat using vertical tows from a depth of 1m. (TIF) [file pone.0214009.s003.tif]

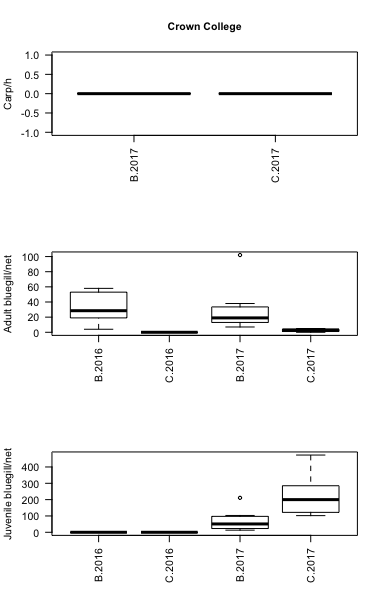

Supplement: S2 Fig — The control lake was invaded by bluegills (note the bluegill presence in the control lake), and was therefore excluded from analyses. (TIFF) [file pone.0214009.s004.tiff]
